# Levels of Organochlorine Pesticides in Brackish Water Fish from Niger River, Nigeria

**DOI:** 10.1155/2018/2658306

**Published:** 2018-06-28

**Authors:** John P. Unyimadu, O. Osibanjo, Joshua O. Babayemi

**Affiliations:** ^1^Nigerian Institute for Oceanography and Marine Research, Victoria Island, Lagos, Nigeria; ^2^Department of Chemistry, University of Ibadan, Nigeria; ^3^Department of Chemical Sciences, Bells University of Technology, Ota, Nigeria

## Abstract

Investigation of the levels of organochlorine pesticides (OCPs) in fish samples was carried out to assess the contamination status of Niger River. Ten different brackish water species of fish (6 samples for each, making a total of 60) were purchased from landing sites at the Delta area of Niger River. These were* Drapane africana, Mochokus niloticus, Chrysichthys nigrodigitatus, Pristipoma jubelini, Vomer septapinis, Pseudotolithus senegalensis, Mugil cephalus, Pseudotolithus elongatus, Sphyraena piscatorum*, and* Lutjanus goreensis*. OCPs were determined using standard methods. Certified reference standards from Accustandard USA were used for the instrument calibration and quantification of OCPs. Twenty OCPs, namely, *α*-HCH, *β*-HCH, *γ*-HCH, *δ*-HCH, endrin, endrin aldehyde, endrin ketone, heptachlor, heptachlor epoxide, aldrin, dieldrin, endosulfan I, endosulfan II, endosulfan sulfate, methoxychlor, *α*-chlordane, *γ*-chlordane, DDE, DDT, and DDT, were identified/quantified using Gas Chromatography (GC) (Hewlett Packard GC 5890 series 11 with electron capture detector). Confirmation was done using Shimadzu GCMS QP2010. The highest concentration of ∑OCPs in the brackish fish samples of the Niger River, 4302±2066 *µ*g/kg fresh weight, with a range of 2237-6368 *µ*g/kg, was detected in* Drapane africana*, while the lowest concentration, 2320±876.4, with a range of 1006-3288 *µ*g/kg, was found in* Mochokus niloticus*. The concentration of total OCP compounds varied markedly amongst the different fish species. The guideline value of 2000 *µ*g/kg fresh weight by WHO/FAO was exceeded and therefore implied potential harmful effects on humans. Since contamination of the fish samples was an indication of contamination of the river, the quality of the water for public water supply should be of concern; and therefore further monitoring is suggested.

## 1. Introduction

Chemical contamination of rivers has been an issue of global concern and continual challenge in developing countries. The modern phenomena of urbanization, population density, and increasing anthropogenic activities are contributing factors. Continual monitoring of chemical pollutants in rivers in advanced countries has been a basis for policy formulation and control of pollution, thereby ensuring safe water bodies. There is, therefore, a need also for regional assessment in Africa in particular, of hazardous substances, specifically persistent organic pollutants (POPs), in rivers.

Amongst the various POPs, organochlorine pesticides (OCPs) have historical use of large amount in Africa, and their presence in food items has been reported [1]. Apart from the fact that fish may be used as an indicator of chemical contaminants in rivers, it is a widely consumed aquatic product in Nigeria; therefore, its safety for consumption as being sourced from major rivers in the country should be assessed.

Onitsha is the largest town along the Niger River with a very important large market. Smoked fish and palm oil/kernel production are common from Warri to Onitsha segments of the river, while sand mining, yam/cassava, and intensive agriculture are dominant in the northern segments. Food crops are cultivated extensively, depending on the existing local ecological zone. A common phenomenon within the entire study area is that special attention is given to trapped fishes in natural ponds and burrowed pits scattered within the extensive floodplains.

The fish communities of the Niger River belong to the Nilo-Sudanian province. In the lower Niger, 160 species have been inventoried in the Kainji Lake [2] amongst which 9 fish families are of economic importance. On the River Benue, about 113 species exist in the Mayo-Kebbi [3]. Until recently, the fauna of the Niger Delta was largely ignored, due to the inaccessibility of the riverine and swampy areas. The Delta has a higher diversity of freshwater fish than any equivalent region in West Africa. In adjacent ecosystems, fish diversity is equivalent to 79 species in the Lagos lagoon of Nigeria [4] and 130 species in the Ebrie Lagoon [5]. As of 2002, there were a total of 311 freshwater fishes in the rivers and lakes in Nigeria. All these species have adapted to the seasonal and interannual variations of the hydrological cycle of the river both in freshwater and in brackish water ecosystems: a succession of favorable and unfavorable environments, appearance, and disappearance of natural habitats.

Our previous studies [6–8] determined the occurrence and distribution of persistent organic pollutants (OCPs and PCBs in particular) in Niger River, and high levels of the pollutants were recorded. It is important to investigate if the pollutants are accumulated in the aquatic organisms, especially those that are usually consumed by humans (fish in particular). This study, therefore, assessed the levels of OCPs in fish from Niger River. Levels in the fish may indicate safety for human consumption.

## 2. Materials and Methods

### 2.1. Fish Sampling

Several prefieldwork activities were undertaken before sampling. These included a reconnaissance of all the proposed sampling locations to determine the easiest and safest access locations. The selected locations along the Niger River were Gurara River (Niger State), Lokoja (Kogi State), Onitsha (Anambra State), and Brass and Nicolas Rivers (Bayelsa State). Fish samples were bought from landing sites in these sampling locations. The fishes sampled were the brackish species and were identified as either demersal, benthic-pelagic, or pelagic. These were* Drapane africana, Mochokus niloticus, Chrysichthys nigrodigitatus, Pristipoma jubelini, Vomer septapinis, Pseudotolithus senegalensis, Mugil cephalus, Pseudotolithus elongatus, Sphyraena piscatorum*, and* Lutjanus goreensis*. Ten different species of fish (6 samples for each, making a total of 60) were purchased. Fish preparation for analysis was as described by Unyimadu et al. [6].

### 2.2. Reagents

All solvents of analytical grade (hexane, acetone, dichloromethane, petroleum spirit, and acetonitrile) were purchased from Merck, Germany, and distilled over 0.5 m packed column (reflux ratio approximately 1:25). The solvent purity was tested by gas chromatographic analyses. Anhydrous granulated sodium sulfate and silica gel 100-200 mesh (Merck, Germany) were cleaned with pure n-hexane by distillation. The external and internal standard were purchased from Restek USA and composed of 1000 *µ*g/ml of the following 20 organochlorine compounds: *α*-HCH, *β*-HCH, *γ*-HCH, *δ*-HCH, endrin, endrin aldehyde, endrin ketone, heptachlor, heptachlor epoxide, aldrin, dieldrin, endosulfan I, endosulfan II, endosulfan sulfate, methoxychlor, *α*-chlordane, *γ*-chlordane, DDE, DDT, and DDT.

### 2.3. Sample Preparation

The details of sample preparation, extraction, extract concentration, lipid determination, extract cleanup, quality control, and quality assurance have been reported elsewhere [7]. In addition to the extract cleanup already reported, the elusion was continued with another 50 ml of 50 + 49.65 + 0.35 DCM/hexane/acetonitrile mixture and the eluate collected in another 100 ml round bottom flask. This fraction called eluate 2 contains endosulfan, dieldrin, endrin, and methoxychlor. The eluates were reduced by volume with a rotary evaporator to 3 ml and solvent exchanged with isooctane and the volume is further reduced to 1 ml in a stream of nitrogen [9].

### 2.4. OCP Analysis

The organochlorine pesticides were screened in the fish samples. The analytical standards (>98% purity) were used to prepare fortification and standard solutions. The extracted samples were subjected to Gas Chromatography (GC) (Hewlett Packard GC 5890 series 11 with electron capture detector) for identification/quantification. Further details, optimization of the instrument, oven temperature programme, flow rate, calibrations, and confirmation (using Shimadzu GCMS QP2010), have been reported elsewhere [7].

## 3. Results and Discussion

### 3.1. Quality Assurance/Quality Control (QA/QC)

Quality control procedures included the analysis of procedural blanks and spiked samples with each set of samples analyzed. Five-point standard curve method was used with r^2^=0.999. The recoveries for the OCPs ranged between 78±2.20 % and 92±2.10 %. To monitor the accuracy of the GC method, an International Atomic Energy Agency (IAEA) Standard Reference Material fish tissue homogenate (IAEA SRM 406) was analyzed with each sample set and the result based on the standard deviation was satisfactory (Table 1).

### 3.2. Biometric Data

The brackish water fishes identified and analyzed were classified into 8 families, namely, Drepaninidae, Bagridae, Haemulidae, Carangidae, Sciaenidae, Mugilidae, Sphyraenidae, and Lutjanidae. All the samples were demersal. The maximum attainable length of the fishes ranged from 45 cm to 120 cm, with the families Sciaenidae, Mugilidae, and Sphyraenidae (e.g.,* Pseudotolithus senegalensis, Mugil cephalus, *and* Sphyraena piscatorum*) growing to a maximum length of 115 to 205 cm, followed by Lutjanidae, 80 cm, Bagridae, Haemulidae, and Sciaenidae (Pseudotolithus elongatus), between 48 cm and 65 cm. The shorter species were Drepanidae and Carangidae: 38 to 40 cm. All the samples were native to the Niger River and belonged to different trophic levels. The trophic levels ranged between 2.4 and 4.4.

At the Gurara sampling, the highest fish weight and lengths were observed in* Clarias gariepinus, *with also the highest fat content.  Table 2 shows the scientific names, family, habitat, maximum length, trophic level, and status of the brackish water fish.

### 3.3. Organochlorine Pesticides in Fish

Monitoring of aquatic pollution can be carried out by means of bioindicator because hydrophobic compounds such as OCPs and PCBs show high affinities for lipids. Assuming that bioconcentration is primarily a result of water-lipid partitioning, the pollutant levels in aquatic biota should reflect concentrations in their environment [10]. Bivalve has been used extensively for this purpose, but fishes have also been selected for monitoring because they concentrate pollutants in their tissues directly from water and also through diet which enables the assessment of transfer of pollutants through the trophic food web [11]. They also generally exhibit low metabolism for organochlorines and consequently should reflect the levels of parent pollutants in the aquatic environment [12].

### 3.4. HCHs

The concentration of HCHs in brackish water fish is shown in Table 3. Technical HCH has been used as a broad spectrum pesticide for an agricultural purpose which has been banned in Nigeria since 1992. Technical HCH consists of four isomers *α*-HCH (60-70 %), *β*-HCH (5-12 %), *γ*-HCH (10-15%), and *δ*-HCH (6-10%), while lindane contains >90 % of *γ*-HCH [13, 14]. *α*- and *β*-HCH were detected in all the brackish fish samples analyzed; *γ*-HCH and *δ*-HCH were detected in 96 % and 98 % of the samples, respectively. The sequence of concentrations in the Niger River brackish fishes were *β*-HCH> *δ*-HCH > *γ*-HCH > *α*-HCH.

The *α*/*γ* ratio has been used to identify the possible HCH source. The ratio of *α*-HCH to *γ*-HCH higher than 3 indicates an input of technical HCH and long-range atmospheric transport and deposition [6, 7]. However, a ratio close to or <1 is characteristic of lindane sources [15, 16]. In this study, the *α*/*γ*-HCH mean ratio was less than 1 in all the brackish fish species studied, indicative of lindane source to these species. Amongst the four HCH isomers, *β*-HCH is easily absorbed by the soil organic matter and more difficult to evaporate from the soil than other HCH isomers [17]. In addition, *α*- and *γ*-HCH can be transformed to *β*-HCH in the environment [18]. Furthermore, the special arrangement of chlorine atoms in the molecular structure of *β*-HCH makes microbial degradation more difficult than the other isomers [19], which may lead to accumulation of *β*-HCH in sediment and subsequently in fish. The profiles of the HCH isomers in sediment (reported elsewhere) and fish were similar signifying that they were from the same sources.

### 3.5. Chlordane

The concentration of chlordane is shown in Table 4. *α*-Chlordane, *γ*-chlordane, heptachlor epoxide, and methoxychlor were detected in all the fish samples analyzed; heptachlor was detected in 98 % of the samples. The sequence of concentration was heptachlor>methoxychlor>heptachlor epoxide > *α*-chlordane>*γ*-chlordane.

The distribution of the chlordane types varied markedly amongst the different brackish fish species with a high concentration of the different isomers identified in different species. The lowest concentration most times was detected in* Pristipoma jubelini*. As reported in Babayemi [1], previous studies in the country showed levels in fish from Lagos lagoon, 0.0793±0.00843 *μ*g/g [20], and similar studies in other countries reported 0.027–0.045 ppb in fish from Tashk Lake in Iran [21]. Maximum residue limits for chlordane in food range between 0.1 and 0.3 mg/kg [1].

### 3.6. DDT

The concentration of DDT and metabolites in brackish water fish is shown in Table 5. DDT, DDE, and DDD were detected in 94 %, 100%, and 92 % of the fish samples, respectively; however, the sequence in the concentration of the metabolites was DDD> DDT >DDE.

The ratios between the parent compound of DDT and its metabolites (DDD and DDE) can be used to identify the possible sources in the aquatic environment [6, 7, 22]. After DDT applications, much of the DDT is slowly converted to DDE and DDD under aerobic and anaerobic conditions, respectively [23, 24]; hence the ratio between the DDT and DDE and DDD is often used as an indication of age (recent to historic) and biotransformation of the DDT [25]. A ratio (DDT/DDD+DDE) much greater than 1 indicates the fresh use of DDT; however, a smaller ratio indicates historical DDT applications [6, 7, 26]. In the present study, the ratio of DDT/DDD+DDE in all the fish samples was less than 1 signifying aged DDT application. The ratio of p'p'-DDT to p'p'-DDE can be used to estimate the existence of technical DDT in recent inputs. A ratio <1 is considered as an aged mixture, while a relatively high (>1) p'p'-DDT/p'p'-DDE indicates DDT in the last 5 years [27, 28].

The ratio of DDD/DDE can reveal the degradation pathways of DDT, since DDE and DDD are aerobic and anaerobic degradation products of DDT, respectively. A ratio of DDD/DDE less than one (<1) shows aerobic degradation and higher than 1 (>1) shows anaerobic degradation [29, 30]. In the fish samples where DDD and DDE were simultaneously detected, the DDD/DDE ratio in the fishes sampled was more than 1 in all cases. These results are indications that the degradation pathways in the Niger River brackish fishes were anaerobic.

### 3.7. Endosulfan

The levels of endosulfan in brackish water fish are shown in Table 5. Endosulfan is a cyclodiene pesticide extensively used throughout the world to control a wide variety of insects and mites. It consists of endosulfan I and II isomers; they are both fairly resistant to photodegradation, but the metabolite endosulfan sulfates are susceptible to photolysis [31]. Because of their high toxicity, technical endosulfan was restricted in many countries. The sequence of occurrence of endosulfan in brackish water fish in this study was endosulfan II > endosulfan sulfate > endosulfan I. Endosulfan I was detected in all the samples analyzed, while endosulfan II and endosulfan sulfate were detected in 92 % and 80 % of the fish samples, respectively.

The distribution of the endosulfan varied markedly amongst the different brackish water fish species with a high concentration of the different isomers identified mostly in* Drapane africana* and the lowest concentration most times detected in* Pristipoma jubelini.* The concentration of endosulfan II was higher than that of endosulfan I, which can be explained by more degradation of endosulfan I in sediment [32]. Endosulfan sulfate, which is a major degradation product of endosulfan, is known to be as toxic as the parent compound.

Endosulfan I and endosulfan II in technical endosulfan account for 70% and 30 %, respectively [32], and the ratio of endosulfan I/endosulfan II in the technical product is about 2.33. Because endosulfan I decomposes more rapidly than endosulfan II in sediment, the ratio of endosulfan I-/endosulfan II <2.33 is used to judge the age of their residues in sediment [6, 7]. The endosulfan I-/endosulfan II isomer ratios in this study in brackish water fish were less than 1 in all the brackish fish samples in which endosulfan I and endosulfan II were simultaneously detected, indicating that there was no recent application of technical endosulfan in the investigated area.

### 3.8. Aldrin and Dieldrin

Aldrin and dieldrin were detected in all the brackish fish samples analyzed (Table 6). The sequence of concentration was aldrin>>>dieldrin in all the samples. The distribution of aldrin and dieldrin varied considerably amongst the different fish species. Generally, environmental releases of aldrin and dieldrin often are directed to the soil. Because of low water solubility and tendency to bind strongly to the soil, both compounds migrate downward very slowly through soil or into surface or groundwater. Also, it is possible that significant volatilization of aldrin/dieldrin might occur, resulting in atmospheric photodegradation. Collectively, these characteristics will foster low levels of aldrin/dieldrin water contamination over comparatively extended periods of time. Dieldrin's extreme polarity results in a high affinity for an organic matter such as animal fats and plant waxes, which could lead to its bioaccumulation in the food chain.

### 3.9. Endrin

Endrin was very well metabolised in the Niger River brackish water fish samples to endrin aldehyde and to endrin ketone. The sequence of concentration in the fish samples was endrin ketone>endrin aldehyde>endrin (Table 6). The only exception to this sequence was in* Drapane africana, *with endrin ketone>endrin >endrin aldehyde. Endrin ketone was detected in 100% of the samples, while endrin aldehyde and endrin were detected in 86 % and 96 % of the samples respectively. Very high levels of endrin ketone were detected in* Pristipoma jubelini, Mugil cephalus, *and* Pseudotolithus elongatus*.

### 3.10. Total OCPs in Brackish Water Fish

The sequence in the concentration of the summation of the different pesticides investigated in fish in this study varied from species to species. DDT, endrin, chlordane, endosulfan, and metabolites were very prominent, compared to HCH, dieldrin, and isomers which occurred at lower concentrations. The sequence in concentration of the organochlorine pesticides was ∑DDT > ∑endrin > ∑chlordane > ∑endosulfan > ∑HCH > ∑dieldrin (Table 7).

The highest concentration of ∑OCPs in the brackish fish samples of the Niger River, 4302±2066 *µ*g/kg fresh weight, with range 2237-6368 *µ*g/kg, was detected in* Drapane africana*, while the lowest concentration, 2320±876.4 with range 1006-3288 *µ*g/kg, was in* Mochokus niloticus*. The concentration of total OCP compounds varied markedly amongst the different fish species of the Niger River with a high concentration of the different isomers identified in different fish species but the lowest concentration most times detected in* Pristipoma jubelini*. The guideline value of 2000 *µ*g/kg fresh weight by WHO/FAO (2000) was exceeded in all the brackish fish samples studied.

### 3.11. Correlation between OCPs, % Fat, and Trophic Levels

There was a very poor correlation between ∑OCPs and % fat in the brackish water fishes, r = 0.048 (p = 0.234), and also very low correlation between trophic level and ∑OCPs, r = 0.045 (p = 0.216). The % fat showed low but positive correlations with ∑HCH, endosulfan sulfate, *δ*-HCH, and *α*-HCH, r = 0.45, 0.35, 0.59, and 0.48, respectively (p = 0.05). The trophic levels show weaker correlations with the OCPs and with ∑HCH, r = 0.25, endrin, r = 0.34, heptachlor, r = 0.24, and *α*-chlordane, r = 0.39, respectively (p = 0.05).

A crucial factor in the pattern and bioaccumulation features of OCPs is interspecies differences [33]. Studying just only five fish species, these authors observed both positive and negative correlations between OCP concentrations and such features as size (length) and fat content of fish species. In the present study, there were ten fish species. The effect of interspecies differences may therefore be expected.

### 3.12. Implications for Human Health

Organochlorine pesticides are toxic and persistent; they bioaccumulate and have potentials for long-range atmospheric transport [34]. The breakdown product (DDE) of DDT leads to breast cancer, infertility in males, liver damage, and nervous system and developmental delay [35]. Heptachlor has the potential to persist in the environment for decades [1] and acute/chronic inhalation exposure may cause nervous disorder or neurological effects [36]. Chlordane may result in distress, tremors, convulsions, and nervous disorder in humans [37]. Endrin in human body fat may constitute neurotoxin [1]. At high concentrations, dieldrin may cause convulsions in humans [38]. Pesticide poisoning had resulted in approximately 200,000 deaths globally, with higher number from developing countries [1, 39].

## 4. Conclusion

The highest concentration of ∑OCPs in the brackish fish samples of the Niger River, 4302±2066 *µ*g/kg fresh weight, with range 2237-6368 *µ*g/kg, was detected in* Drapane africana*, while the lowest concentration, 2320±876.4 with range 1006-3288 *µ*g/kg, was in* Mochokus niloticus*. The concentration of total OCP compounds varied considerably amongst the different fish species. The guideline value of 2000 *µ*g/kg fresh weight by WHO/FAO was exceeded and therefore implied potential harmful effects on humans. Since contamination of the fish samples was an indication of contamination of the river, the quality of the water for public water supply should be of concern; and therefore further monitoring is suggested.

## Figures and Tables

**Table 1:** Certified reference material results (*µ*g/kg dw).

Compound	SRM Fish homogenate		
	SRM Value	This Study	SD
*α*-HCH	0.79	0.61	0.09
*β*-HCH	0.75	0.82	0.04
*γ*-HCH	0.27	0.33	0.08
p'p'-DDT	3.00	4.50	0.75
p'p'-DDE	-	-	-
p'p'-DDD	-	-	-
Heptachlor	-	-	-
Heptachlor epoxide	0.99	0.88	0.06
Aldrin	-	-	-
Dieldrin	3.50	3.10	0.20
Endrin	1.90	1.45	0.23
Endosulfan I	3.50	3.08	0.21
Endosulfan II	1.40	1.09	0.15
Endo. sulfate	3.60	3.12	0.24
*α*-Chlordane	-	-	-
*γ*-Chlordane	0.70	0.65	0.03

**Table 2:** Brackish fish, scientific names, family, habitat, maximum length, trophic level, and status.

Names	Family	Habitat	*∗*Max length	*∗*Trophic level	Status
*Drepane africana *	Drepanidae	Demersal	45	3.1	Native
*Mochokus niloticus*	-	Demersal	-	-	Native
*Chrysichthys nigrodigitatus*	Bagridae	Demersal	65	3.2	Native
*Pomadasys jubelini *	Haemulidae	Demersal	60	3.3	Native
*Vomer septapinis*	Carangidae	Demersal	38	4.1	Native
*Pseudotolithus senegalensis*	Sciaenidae	Demersal	114	3.8	Native
*Mugil cephalus*	Mugilidae	Demersal	120	2.4	Native
*Pseudotolithus elongatus*	Sciaenidae	Demersal	48	4.1	Native
*Sphyraena piscatorum*	Sphyraenidae	Demersal	205	4.1	Native
*Lutjanus goreensis*	Lutjanidae	Demersal	80	4	Native

*∗*Source: www.fishbase.org.

**Table 3:** Organochlorine pesticide (HCH) levels in brackish fish (*µ*g/kg fresh weight).

Fish Species/ Number	*α*-HCH	*β*-HCH	*γ*-HCH	*δ*-HCH
*Drapane Africana *	36.73±28.85 (7.88-65.58)	142±56.74 (85.52-199.0)	89.37±26.63 (62.75-166.0)	89.42±29.58 (59.84-119)
*Mochokus Niloticus *	13.15±9.27 (0.72-26.43)	64.93±80.53 (2.79-226.0)	30.90±27.36 (4.97-85.63)	53.87±25.75 (5.96-81.32)

*Chrysichthys nigrodigitatus*	35.04±3.69 (29.50-39.32)	93.71±44.18 (33.7-160)	29.53±30.80 (BDL-75.74)	119±100 (39.29-271)
*Pristipoma jubelini*	23.91±12.33 (10.58-38.55)	42.39±10.34 (25.21-52.56)	9.08±4.33 (2.33-9.86)	73.85±21.89 (48.36-99.45)
*Vomer Septapinis*	36.47±8.21 (20.83-54.26)	101±67.62 (43.14-271)	25.11±24.46 (BDL-64.68)	96.06±56.34 (19.24-197)
*Pseudotolithus senegalensis*	34.02±1.65 (16.35-54.26)	64.46±21.33 (46.80-163.0)	39.43±25.25 (25.43-46.7)	88.71±47.29 (19.24-125.0)

*Mugil cephalus*	55.26±5.70 (44.65-66.29)	148±105 (59.96-360.0)	49.30±27.34 (19.71-104.0)	114±81.34 (16.9-248.0)
*Pseudotolithus elongatus*	34.53±23.32 (2.34-61.91)	151±130 (4.02-490.0)	32.67±32.24 (BDL-74.03)	93.75±94.12 (BDL-282.0)
*Lutjanus goreensis *	38.5±17.2 (22.2-56.3)	127.4±14.0 (114.8-140.0)	79.0±8.00 (70.0-87.5)	82.5±49.3 (33.5-132.0)
*Sphyraena piscatorum*	34.1±13.1 (18.1-52.6)	122.0±9.00 (109.5-135.8)	74.8±4.67 (65.0-82.0)	78.4±45.3 (28.2-129.0)

**Table 4:** Chlordane levels in brackish fish *µ*g/kg fresh weight (fw) (n=6).

Fish Species/No	*γ*-Chlordane	*α*-Chlordane	Heptachlor	Heptachlor epoxide	Methoxychlor
*Drapane Africana (n=6)*	87.68±16.32 (71.4104.0)	123±50.30 (73.4-174.0)	400.0±35.0 (365.0-435.0)	71.1±22.5 (48.6-93.6)	154±91.51 (62.9-246.0)
*Mochokus Niloticus (n=6)*	40.05±27.58 (13.6-95.3)	33.55±30.26 (7.58-94.1)	112.1±106.0 (BDL-235.0)	98.8±71.6 (40.5-242.0)	88.76±23.73 (46.7-118.0)
*Chrysichthys nigrodigitatus*(n=6)	60.16±26.85 (19.9-98.7)	45.10±22.09 (13.8-78.3)	145.0±39.3 (109.0-204.0)	113.0±66.9 (13.0-175.0)	77.87±31.42 (41.9-125.0)
*Pristipoma jubelini* (n=6)	9.14±3.11 (3.24-15.7)	37.62±8.45 (24.8-40.6)	120.0±45.23 (88.2-188.2)	19.1±4.33 (24.8-40.6)	107.0±34.2 (68.8-116.0)
*Vomer Septapinis* (n=6)	50.21±10.55 (23.83-65.08)	59.60±25.31 (26.28-92.60)	179.0±60.2 (108.0-330.0)	70.92±25.35 (42.5-123)	100. ±23.31 (70.32-156)
*Pseudotolithus senegalensis* (n=6)	40.59±16.76 (50.91-69.1)	58.09±31.81 (35.3-44.4)	137.0±29.5 (274.0-330.0)	48.27±5.77 (12.3-150.0)	76.03±5.72 (156.0-231.0)
*Mugil cephalus* (n=6)	43.77±14.59 (24.94-72.9)	61.84±37.58 (25.7-61.8)	262.0±108.0 (61.6-468.0)	154.0±33.0 (111.0-197.0)	107±47.47 (44.3-202.0)
*Pseudotolithus elongatus* (n=6)	40.23±19.31 (1.62-74.19)	61.57±47.22 (13.8-156.0)	254±197.0 (12.5-623.0)	107±86.16 (1.80-201.0)	142±87.47 (29.3-317.0)
*Lutjanus Goreensis (6)*	43.6±2.78 (40.9-45.2)	61.5±6.40 (55.3-68.1)	509.9±240.2 (244.3-809.5)	123.7±96.4 (33.0-215.6)	410.8±317.2 (73.8-430.9)
*Sphyraena piscatorum(6)*	38.3±1.28 (35.8-40.6)	55.4±1.68 (50.1-62.6)	410±150 (145.6-760.5)	118.5±88.4 (25.7-209.5)	390.2±218.4 (64.8-740.8)

**Table 5:** Organochlorine pesticides (DDT and endosulfan) concentrations (*µ*g/kg fresh weight) in brackish water fish.** **

Fish Species/No	p′p'DDD	p′p'DDE	p'p'DDT	Endosulfan I	Endosulfan II	Endosulfan Sulfate
*Drapane Africana (n=6)*	1232±1087 (145.0-2320)	94.5±9.54 (84.9-104.0)	234.0±173.0 (61.2-408.0	68.7±21.1 (47.6-89.8)	493.0±333.0 (160.0-826)	233.0±29.0 (204.0-262)
*Mochokus Niloticus (n=6)*	654.0±584.0 (BDL-1634	81.6±52.4 (24.4-134.0)	191.0±176.0 (BDL-455.0)	32.5±12.9 (6.75-43.2)	337.0±379.0 (37.4-1096)	49.1±63.9 (BDL-177.0)
*Chrysichthys nigrodigitatus* (n=6)	244.0±244 (BDL-612.0)	100.0±43.6 (50.9-166.0)	133.0±115.0 (15.1-307.0)	34.3±21.1 (15.7-65.9)	254.0±230.0 (46.6-600.0)	47.2±31.5 (BDL-79.4)
*Pristipoma jubelini* (n=6)	95.7±25.1 (52.2-111.0)	63.0±34.1 (28.7-118.0)	90.4±43.4 (28.7-118.0)	25.4±15.1 (12.3-40.6)	95.7±25.1 (54.2-111.0)	2.33±0.97 (BDL-5.45)
*Vomer Septapinis* (n=6)	476.0±407.0 (67.3-1494)	65.1±21.6 (32.5-118)	97.5±89.2 (BDL-304.0)	42.5±21.4 (14.3-82.5)	386.0±272.0 (37.2-1022)	230.0±204.0 (BDL-741)
*Pseudotolithus senegalensis* (n=6)	859.0±635.0 (67.3-2800)	49.2±16.8 (44.8-67.2)	168.0±135.0 (35.9-558.2)	48.4±34.1 (17.2-29.1)	529.0±492.0 (152.0-1830)	119.56±26.1 (25.6-202.2)
*Mugil cephalus* (n=6)	473.0±267.0 (BDL-860.0)	84.1±43.4 (8.34-132.0	133.0±125 (36.0-384.0)	25.5±12.6 11.5-42.2	222.0±168.0 (BDL-559.0)	83.5±94.7 (BDL-273.0)
*Pseudotolithus elongatus* (n=6)	901.0±523.1 (223-1987)	52.6±21.6 (11.6-83.1)	245.0±201.0 (21.6-492)	27.3±12.6 (2.00-47.9)	508.0±413.0 (40.5-1328)	6.25±9.58 (BDL-25.0)
*Lutjanus goreensis(n=6)*	141.0±45.0 78.0-164.5	42.3±27.9 14.1-69.7	31.7±21.9 BDL-68.9	45.5±6.58 38.0-56.9	196.5±18.5 178.5-210.7	173.6±138.5 36.9-300.4
*Sphyraena piscatorum (n=6)*	130.6±36.8 67.9-153.8	38.3±21.9 7.28-60.5	28.3±17.50 BDL-56.30	40.1±3.80 34.5-50.1	186.5±9.00 168.4-200.3	164.8±132.2 28.6-297.4

**Table 6:** Concentration (*µ*g/kg fresh weight) of cyclodienes in brackish fish.

Fish Species/No	Endrin	Endrin aldehyde	Endrin Ketone	Aldrin	Dieldrin
*Drapane Africana (n=6)*	108.0±40.3 (68.4-149.0)	42.0±4.20 (BDL-42.20)	310.0±211.0 (99.3-522.0)	258.0±112.0 (147.0-370.0)	25.7±8.68 (17.0-34.4)
*Mochokus Niloticus (n=6)*	45.6±29.7 (22.5-105.0)	167.2±202.0 BDL-584.0	324.0±99.5 (139.0-480.0)	143.0±110.0 (3.31-355)	26.5±13.9 (10.8-54.3)
*Chrysichthys nigrodigitatus* (n=6)	77.4±47.5 (6.05-131.0)	205.0±120.0 (24.7-304.0)	310.0±116.0 (166.0-484)	165.0±81.1 (56.1-287.0)	27.7±13.8 7.04-40.72
*Pristipoma jubelini* (n=6)	38.2±15.3 (20.0-56.9)	971.0±110.0 (720.0-988.0)	748.0±122.0 (521.0-829.0)	36.9±11.3 (18.2-50.6)	27.6±12.1 (18.6-44.4)
*Vomer Septapinis* (n=6)	46.6±38.2 (BDL-112.0)	99.3±95.7 (BDL-236.0)	297.0±106.0 (124.0-453)	191.0±81.77 (35.94-307)	26.5±8.82 (15.0-41.9)
*Pseudotolithus senegalensis* (n=6)	38.34±38.3 15.9-112.0	229.4±29.36 (216.0-236.0)	351.0±57.5 (162.0-453.0)	88.9±53.0 214.0-321.0	37.6±4.38 14.2-45.0
*Mugil cephalus* (n=6)	54.9±31.0 BDL-117	14.5±21.7 BDL-57.9	494.0±384.0 (81.9-1263)	321.0±73.8 194.0-423.0	24.3±15.5 6.57-50.1
*Pseudotolithus elongatus* (n=6)	74.6±46.5 8.22-133.0	16.1±24.8 BDL-64.4	437.0±167.4 127.0-725.4	233.1±124.0 21.81-455	24.2±15.6 4.68-55.4
*Lutjanus goreensis (n=6)*	68.3±22.5 47.0-92.6	146.4±45.8 81.2-172.8	210.7±114.0 96.0-324.0	404.1±32.5 398.1-450.5	28.1±3.40 21.2-33.4
*Sphyraena piscatorum (n=6)*	64.2±18.1 40.1-86.0	138.9±36.4 76.2-168.1	198.0±111.2 88.4-310.2	396.4±28.1 345.0-420.1	26.8±2.30 18.4-28.6

**Table 7:** Sum of organochlorine pesticides concentrations (*µ*g/kg fresh weight) in brackish water fish.

Fish Species/No	∑HCH	∑Chlordane	∑Endrin	∑Endosulfan	∑DDT	∑Dieldrin	∑OCPs
*Drapane Africana (n=6)*	357.0±82.5 275.0-671.0	836.3±215.3 621.4-1052	461.1±210.4 251.0-671.3	794.5±383.2 412.3-1177	1561±1271 291.6-2832	284.2±103.2 181.2-387.5	4302±2066 2237-6368
*Mochokus Niloticus (n=6)*	158.0±128.0 29.3-416.0	350.5±241.3 83.7-751.3	448.5±360.3 38.0-1169	418.3±360.9 96.1-1139	927.3±762.1 24.4-1949	170.2±122.5 22.63-409.0	2590±1363 2006-3421
*Chrysichthys nigrodigitatus* (n=6)	277.0±126.0 155.0-467.0	407.3±63.8 317.2-503.6	592.1±263.8 197.2-866.5	336.2±190.2 124.0-621.3	358.2±188.2 188.3-641.4	195.0±88.6 63.2-328.9	2320±876.4 1006-3288
*Pristipoma jubelini* (n=6)	107.3±34.2 68.9-116.0	293.4±88.3 212.4-324.6	1757±211.8 1578-1903	96.1±32.4 56.3-112.6	250.3±67.6 187.3-269.4	64.7±43.2 48.4-99.6	2610±233.6 1978-3234
*Vomer Septapinis* (n=6)	259.8±88.5 131.0-397.0	442.3±116.2 314.2-704	443.9±187.6 124.6-801.2	660.2±359.1 51.5-1104	639.6±496.3 148.4-1864	217.3±73.0 69.1-334.0	2681±655.4 1440-4140
*Pseudotolithus senegalensis* (n=6)	226.0±95.5 167.0-330.0	360.1±7.50 704.2-759.5	419.2±125.6 394.2-801.3	577.2±526.3 238.3-371.2	1077±786.5 148.0-4031	126.0±57.4 229.1-335.0	2790±1350 2421-2490
*Mugil cephalus* (6)	393.0±159.0 181.0-712.0	614.3±73.5 51.2-737.2	564.3±378.2 134.7-1321	331.7±153.2 95.4-571.0	690.2±362.5 168.0-1078	346.2±78.0 205.2-473.2	2942±723.0 2027-3858
*Pseudotolithus elongatus* (n=6)	311.0±216.0 33.0-809.0	603.2±213.1 46.8-866.1	528.2±123.8 300.2-743.2	617.3±412.2 96.3-1356	1189±602.1 295.3-2355	258.4±176.3 26.49-510	3451±1456 1921-5948
*Lutjanus goreensis (n=6)*	321.8±86.5 248.0-431.2	1116±820.5 750.8-2186	420.4±231.3 286.2-580.2	450.3±180.2 261.1-530.2	360.3±99.2 260.8-390.5	460.8±35.0 410.8-520.1	3148±1126 1196-4180
*Sphyraena piscatorum (n=6)*	300.9±78.6 230.6-420.4	930.2±420.4 330.1-1920	399.2±140.1 230.8-490.2	410.6±120.3 230.1-515.4	318.3±68.2 250.3-350.5	420.8±22.6 390.8-450.4	3000±950.3 1345-3948
